# Will healthcare workers improve infection prevention and control behaviors as COVID-19 risk emerges and increases, in China?

**DOI:** 10.1186/s13756-020-00746-1

**Published:** 2020-06-11

**Authors:** Xiaoquan Lai, Xuemei Wang, Qiuxia Yang, Xiaojun Xu, Yuqing Tang, Chenxi Liu, Li Tan, Ruying Lai, He Wang, Xinping Zhang, Qian Zhou, Hao Chen

**Affiliations:** 1 Tongji Hospital, Tongji Medical College, Huazhong University of Science and Technology, Wuhan, China; 2 Present address: School of Medicine and Health Management, Tongji Medical School, Huazhong University of Science and Technology, No.13 Hangkong Rd, Wuhan, Hubei Province China; 3 First Affiliated Hospital of Gannan Medical University, Ganzhou, China

**Keywords:** COVID-19, Infection prevention and control behaviors, Outbreak risk, Contact with confirmed and suspected patients, High-risk department, Affected area

## Abstract

**Background:**

COVID-19 arise global attention since their first public reporting. Infection prevention and control (IPC) is critical to combat COVID-19, especially at the early stage of pandemic outbreak. This study aimed to measure level of healthcare workers’ (HCW’) self-reported IPC behaviors with the risk of COVID-19 emerges and increases.

**Methods:**

A cross-sectional study was conducted in two tertiary hospitals. A structured self-administered questionnaire was delivered to HCWs in selected hospitals. The dependent variables were self-reported IPC behavior compliance; and independent variables were outbreak risk and three intent of infection risk (risk of contact with suspected patients, high-risk department, risk of affected area). Chi-square tests and multivariable negative binomial regression models were employed.

**Results:**

A total of 1386 participants were surveyed. The risk of outbreak increased self-reported IPC behavior on each item (coefficient varied from 0.029 to 0.151). Considering different extent of risk, HCWs from high-risk department had better self-reported practice in most IPC behavior (coefficient ranged from 0.027 to 0.149). HCWs in risk-affected area had higher self-reported compliance in several IPC behavior (coefficient ranged from 0.028 to 0.113). However, HCWs contacting with suspected patients had lower self-reported compliance in several IPC behavior (coefficient varied from − 0.159 to − 0.087).

**Conclusions:**

With the risk of COVID-19 emerges, HCWs improve IPC behaviors comprehensively, which benefits for better combat COVID-19. With the risk (high-risk department and affected area) further increases, majority of IPC behaviors achieved improvement. Nevertheless, under the risk of contact with suspected patients, HCWs show worse IPC behaviors. Which may result from higher work load and insufficient supplies and resources among these HCWs. The preparedness system should be improved and medical assistance is urgently needed.

## Background

A mysterious infectious illness has been epidemic in Wuhan, China, which showed an alarming spread speed since their first public reporting on 31st, December 2019. According to epidemiological survey, SARS-CoV-2 was identified as pathogen of this epidemic, which is a new strain that has not been previously identified in humans.

As the epidemic further intensifies, human-to-human transmission has been eventually confirmed, and increasing confirmed and suspected cases are being identified with the use of nucleic acid detection kit. Worse still, spread of COVID-19 is getting more critical with the arrival of Spring Festival, leading to over 1 billion people flows during the Spring Festival travel rush, among which 5 million people left Wuhan. Up to mid-February, a total of 56,568 confirmed cases and 8969 suspected cases have been officially reported in China, among whom 11,053 patients were in critical conditions and 1524 have died. A global attention has been drawn just like the SARS-COV outbreak in 2002–03, which caused 774 cumulative deaths. Under such critical circumstances, World Health Organization (WHO) urgently reconvened the second emergency committee and eventually declared that the outbreak of COVID-19 constituted a Public Health Emergency of International Concern (PHEIC).

As a key member combating COVID-19, healthcare workers (HCWs) play an essential role in containing outbreak and alleviating the increasing infection risk, who are also being exposed to the infected danger. For instance, the number of HCW affected during the COVID-19 outbreak was up to 1706 cases, accounting for approximately 20% of all infection cases. According to official report, 15 HCWs have been identified with COVID-19 at the early stage of outbreak, which would be even worse with increasing severe outbreak. To effectively reduce the risk of COVID-19 transmission in healthcare institutions and standardize HCWs’ behaviors, Chinese government required HCWs to strictly implement standardized preventive measures and strengthen protective measures against droplet isolation, contact isolation and air isolation. Recommended IPC measures by WHO include hand hygiene, medical mask, use of personal protective equipment (PPE), single or cohort patients, sterilization of patient-care equipment and linen etc. [1].

There are salient evidences shown that proper IPC measures during outbreak management could remarkably change the course of the outbreak [2]. However, the current IPC behaviors are far from optimal. A survey among HCWs during Lassa Fever outbreak showed that none met the minimum standard for IPC during the first contact with possible Lassa Fever cases [3]. Actually, policy rule is not the only determinant of IPC behaviors of HCWs, as optimal IPC behaviors are influenced by many factors. Outbreak, contact with confirmed and suspected patients, key clinical departments (such as intensive care unit and emergency department) are critical risk factors in the pandemic outbreak and always cited as important causes of high healthcare-associated prevalence worldwide [4–6]. Other influencing factors associated with HCWs’ IPC behaviors include years of experience, preparedness etc. [5].

At the early stage of pandemic outbreak, improving IPC behaviors of HCWs is of great significance, which could help guide evidence-based optimal IPC behaviors to combat future large-scale outbreak. However, studies investigating IPC behaviors at the early stage of outbreak have not been found so far, especially those concerning behaviors and key risk determinants of IPC. To bridge previous gaps, two research questions were put forward: (1) Will HCWs improve their IPC behaviors as the risk of COVID-19 emerges? (2) Will HCWs further improve their IPC behaviors as the risk of COVID-19 increases? An epidemiologic study focusing on self-reported IPC behaviors before and after COVID-19 was conducted among HCWs in Wuhan city, Hubei Province and Ganzhou city, Jiangxi Province at the early stage of pandemic outbreak. This study aimed to evaluate the change of HCWs’ self-reported IPC behaviors as the risk of COVID-19 emerges and increases, so as to provide evidence-based IPC management for broader target populations to better combat COVID-19.

## Methods

### Study settings

An institution based cross-sectional study was conducted from 15th to 17th January 2020 at 2 tertiary hospitals, during the period the transmission route was officially reported to be limited human-to-human transmission. The two hospital locate in Wuhan city, Hubei province, while the other locates in Ganzhou City, Jiangxi Province.

The hospital surveyed in Wuhan has 6000 inpatient beds proving 6,317,152 outpatient and 272,002 inpatient services mainly for its inhabitants in its catchment area, which was one of the leading hospitals accepted patients with COVID-19 since the beginning of the outbreak in Mid-December 2019.

The hospital surveyed in Ganzhou has 3000 inpatient beds and services 1,303,196 outpatients and 109,225 inpatients in 2019, and is also the top hospital in Ganzhou, Jiangxi, which is the neighbor of Hubei province (hospital not in affected area). When we conducted our study, no patient was confirmed COVID-19 outside Hubei province. Wuhan and Ganzhou had over 11 and 9.8 million population covering approximately 8494 and 39,380 km^2^, respectively. Both cities are located in central China and ranges in the middle level regarding economic development of all cities in China.

### Data collection

A structured self-administered questionnaire was used to collect the data. Study participants in selected hospitals who were willing to take part in the study were asked to return questionnaires to the data collectors. The questionnaires were collected, and incomplete questionnaire was taken back to the respondent for completion as much as possible.

The questionnaire was developed based on the guideline issued by hospital in affected area, which was consistent with “Infection prevention and control during health care when 2019-nCoV infection is suspected Interim guidance” proposed by WHO (released after design). According to this guideline, the standardized self-reported IPC measures recommended by HCWs included the early recognition and immediate placement, the hand hygiene, use of personal protective equipment (PPE) depending on risk, sterilization of patient-care equipment and linen for all patients and contact and airborne precautions for suspected COVID-19.

### Measurement and hypothesis

#### Dependent variables

According to the guideline proposed by WHO, twelve items were developed to capture the degree of HCWs’ self-reported behavior to these recommended measures. Five items were related to the moments of hand hygiene behaviors, including (1) before direct contact with patients; (2) before aseptic operation; (3) after exposed to patient’s body fluid; (4) after direct contact with patients; (5) after exposed to patients’ surroundings. Four items were related to use of PPE after exposed to high risk patients including use of masks, gloves, goggles and gowns. One item was related to proper isolation for confirmed and suspected cases. The other two items were related to terminal disinfection of each patient bed and reporting to superior of confirmed and suspected cases (Table 1).

**Table 1:** Description of main variable

Variable name	Description
**Independent variable**	
***Outbreak risk***	1 = yes/exist, 0 = no/not exist
***Extent of risk***	
Risk of contact with confirmed and suspected patients	1 = yes/contacted, 0 = no/not contacted
High-risk Department	1 = High-risk department (department of respiratory medicine, infectious disease, emergency and general intensive care unit) 0 = Not high-risk department
Risk of affected area	1 = yes/Wuhan, 0 = no/Ganzhou
**Dependent variable**	
1 Compliance of hand hygiene: Overall hand hygiene, and it’s five moment, including before touching a patient, before aseptic procedure, after body fluid exposure risk, after touching a patient, after touching patient surroundings	
2 Compliance of use of personal protective equipment: Overall personal protective equipment, and use of mask, use of glove, use of goggle, use of gown	
3 Compliance of appropriate patient placement	
4 Compliance of terminal disinfection	
5 Compliance of report to superior	

For each item, the respondents were asked how many times they had complied with the recommended IPC guideline during the last 10 corresponding operations (0–10 points). To compare how these IPC behaviors changed with the outbreak of COVID-19, HCWs’ self-reported compliance score after the outbreak and before the outbreak on each item were collected. They served as dependent variables in this study. We chose self-reported compliance score rather than direct observation to prevent the risk of infection in observers.

#### Independent variables

Except for outbreak risk mentioned above (measure as after the outbreak or before the outbreak), three independent variables were included in this study to reflect the extent of infection risks, i.e. whether the respondents were exposed to confirmed and suspected patients (defined as flu-like cases with body temperature above 38 °C and sore throat or cough), whether the respondents worked in high-risk departments (defined as department of respiratory medicine, infectious disease, emergency and general intensive care unit), whether HCWs worked in Wuhan City as the risk of affected area. Socio-demographic characteristics (including gender, career type, age, work year, education degree, working title, work load) were also recorded. The work load was measured using self-reported five-point Likert scale (very low to very high) (Table 1).

$$ \mathrm{Self}-\mathrm{reported}\ \mathrm{compliance}\ \mathrm{of}\ \mathrm{IPC}\ \mathrm{measure}=\frac{number\ of\ self- reported\ behaviors\ conforming\ to\ guidelines}{total\ number\ of\ self- reported\ behaviors} $$

The basic hypothesis of this study are as follows:

- **Hypothesis1:** As the risk of COVID-19 emerges, HCWs will improve their IPC behaviors.
- **Hypothosis2:** As the risk of COVID-19 increases, HCWs will further improve their IPC behaviors.

### Data analysis

Descriptive statistics were used to describe the demographic characteristics of participants. Means of self-reported compliance score on each item after and before the outbreak were calculated separately. Chi-square test was then conducted to analyze the differences of self-reported compliance scores between different time frame (indicating the exist of outbreak risk or not), and the differences of self-reported compliance scores after outbreak in different extent of risk, including exposure state (indicating risk of contact with confirmed and suspected patients or not), working department (indicating high-risk department or not) and district (indicating risk of affected area or not).

To further establish the associations between each self-reported compliance item score and the potential risk factors, we performed multivariable negative binomial regression models. To estimate the effect of outbreak risk, we included each self-reported compliance item score before and after outbreak as dependent variables and included the different time frame (indicating the exist of outbreak risk or not) and socio-demographic characteristics of investigated HCWs as independent variables. Clustered sandwich estimator was used to control the individual intragroup correlation due to the inclusion of both before and after compliance. Because the extent of risk might only affect the compliance after the outbreak is issued, we included each compliance item score after outbreak as dependent variables and different exposure state (indicating risk of contact with confirmed and suspected patients or not), different working department (indicating high-risk department or not) and different district (indicating risk of affected area or not) and the socio-demographic characteristics as dependent variables to estimate the effect of the extent of risk. Negative binomial regression was used because the dependent variable in the study was highly skewed and over-dispersed [7]. For the variables of socio-demographic characteristics, we treated gender and career as categorical variables and treated age and work year as continuous variables. LR/ BIC/ AIC tests were performed to determine the ordinal variables (degree, title, workload) as continuous or categorical [8]. All the analyses were performed using Stata 15.0 (StataCorp LP, College Station, TX, USA).

## Results

### Characteristics of participants’ risk and demographic

A total of 1581 participants were surveyed, with 1386 questionnaires included and 195 questionnaires excluded for the miss in the self-reported behavior of IPC measures before or after the outbreak risk, indicating that response rate was 87.67%. More than a quarter of HCWs had the experience of contacting the confirmed and suspected patients (26.4%), while 19.28% HCWs worked in high-risk department and more than one-half HCWs were from affected area (55.41%). The mean age and work year were 30.88 and 7.71, respectively. Most of them were female (77.2%), undergraduate degree (63.4%), junior title (49.6%) and high work load (47.4%) (Table 2).

**Table 2:** Risk and demographic characteristics of participants

Variable name	mean (SD ^b^) / No. (%)
**Risk of contact with confirmed and suspected patients**^**a**^	
Contacted	314 (26.4)
Not contacted	875 (73.6)
**Department of high risk**^**a**^	
High-risk department	262 (19.28)
Not high-risk department	1097 (80.27)
**Risk of affected area**	
In affected area	768 (55.41)
Not in affected area	618 (44.59)
***Demographic characteristics***	
**Gender**^**a**^	
Male	314 (22.8)
Female	1061 (77.2)
**Career**^**a**^	
Doctor	477 (35)
Nurse	886 (65)
**Age**^**a**^**,** Mean (SD)	30.88 (6.30)
**Work year**^**a**^**,** Mean (SD)	7.71 (6.37)
**Education degree**^**a**^	
Below junior college	5 (0.4)
junior college	123 (9)
Undergraduate	863 (63.4)
Master	226 (16.6)
Doctor	145 (10.6)
**Title**^**a**^	
Not evaluate	119 (9.4)
Junior	625 (49.6)
Intermediate	412 (32.7)
Deputy senior	79 (6.3)
Senior	26 (2.1)
**Work load**^**a**^	
Very low	4 **(**0.3**)**
Low	30 **(**2.4**)**
Neutral	442 **(**35.3**)**
High	594 **(**47.4**)**
Very high	183 **(**14.6**)**

a: A total of 11 HCWs missed gender, 23 missed career, 31 missed age, 58 missed work year,24 missed education degree, 125 missed title, 133 missed work load, 197 missed risk of contact with confirmed and suspected patients and 27 missed department

b: SD refers to Standard Deviation

### Univariate analysis in self-reported compliance under different COVID-19 risk

The self-reported compliance of all IPC measures after COVID-19 outbreak risk existed was significantly higher than that before (*p* < 0.001), which supported hypothesis1. The self-reported compliance of use of goggle and grown improved a lot, whereas the self-reported compliance was still below 90%. As for hand hygiene, the self-reported compliance before touching a patient and after touching patient surrounding was relatively lower than others, even after COVID-19 outbreak. The effect of exist of risk was determined, whereas the effect of risk in different extent was mixed. HCWs in high-risk department were associated with higher self-reported compliance in nine measures including hand hygiene before touching a patient (*p* < 0.001), after body fluid exposure risk (*p* = 0.036), after touching a patient (*p* = 0.027), after touching patient surrounding (*p* = 0.001) and overall hand hygiene (*p* < 0.001), appropriate patient placement (*p* < 0.030), use of goggle (*p* < 0.001), use of gown (*p* = 0.013), use of PPE (*p* < 0.001). HCWs with the risk of affected area showed higher self-reported compliance of all the IPC measures, which supported hypothesis2. However, HCWs with the risk of contact with confirmed and suspected patients had significantly lower compliance in all the measures, which was opposite to hypothesis2 (Table 3).

**Table 3:** Self-reported compliance in HCWs under different COVID-19 risk

	Outbreak risk			Extent of risk								
				Risk of contact with the confirmed and suspected patients			High-risk department			Risk of affected area		
	Yes	No	*p*-value	Yes	No	*P*-value	Yes	No	*P*-value	Yes	No	*P*-value
Before touching a patient	0.937	0.842	< 0.001***	0.925	0.936	0.046**	0.953	0.934	< 0.001***	0.942	0.931	0.007***
Before aseptic procedure	0.981	0.946	< 0.001***	0.973	0.982	0.003***	0.978	0.982	0.272	0.985	0.975	< 0.001***
After body fluid exposure risk	0.989	0.960	< 0.001***	0.980	0.992	< 0.001***	0.992	0.987	0.036**	0.993	0.983	< 0.001***
After touching a patient	0.966	0.905	< 0.001***	0.954	0.969	< 0.001***	0.974	0.965	0.027**	0.977	0.953	< 0.001***
After touching patient surroundings	0.929	0.843	< 0.001***	0.911	0.931	< 0.001***	0.943	0.925	0.001***	0.942	0.913	< 0.001***
**Overall hand hygiene**	0.962	0.903	< 0.001***	0.812	0.928	< 0.001***	0.965	0.954	< 0.001***	0.963	0.947	< 0.001***
**appropriate patient placement**	0.960	0.900	< 0.001***	0.932	0.973	< 0.001***	0.938	0.930	0.030**	0.928	0.932	< 0.001***
Use of mask	0.987	0.946	< 0.001***	0.982	0.989	0.002***	0.987	0.986	0.633	0.991	0.981	< 0.001***
Use of glove	0.957	0.878	< 0.001***	0.932	0.964	< 0.001***	0.961	0.956	0.278	0.970	0.942	< 0.001***
Use of goggle	0.800	0.687	< 0.001***	0.702	0.820	< 0.001***	0.826	0.794	< 0.001***	0.828	0.764	< 0.001***
Use of gown	0.829	0.728	< 0.001***	0.727	0.854	< 0.001***	0.847	0.826	0.013**	0.867	0.782	< 0.001***
**use of personal protective equipment**	0.895	0.813	< 0.001***	0.760	0.846	< 0.001***	0.891	0.868	< 0.001***	0.892	0.848	< 0.001***
**terminal disinfection**	0.957	0.905	< 0.001***	0.945	0.962	< 0.001***	0.954	0.958	0.409	0.973	0.938	< 0.001***
**Report to superior**	0.970	0.918	< 0.001***	0.948	0.979	< 0.001***	0.965	0.971	0.151	0.983	0.953	< 0.001***

Significance: ***p < 0.01, **p < 0.05, *p < 0.10

### Multivariable analysis in compliance of major IPC measures under different COVID-19 risk

All the significant variables were shown. As the LR/ BIC/ AIC tests in all the model were examined with *p* > 0.05, the ordinal variables (education degree, title, workload) were included as continuous variables in the model. As the coefficients of outbreak risk were positive, the exist of outbreak risk significantly increased all the self-reported compliance of all the IPC measures including overall hand hygiene (Coef. = 0.070, *p* < 0.001), overall use of PPE (Coef. = 0.109, *p* < 0.001), appropriate patient placement (Coef. = 0.063, *p* < 0.001), terminal disinfection (Coef. = 0.059, *p* < 0.001) and report (Coef. = 0.059, *p* < 0.001). As for the extent of risk, the positive coefficient indicated that the high-risk department also was significantly associated with higher self-reported compliance of IPC measures in the hand hygiene after touching patient surroundings (Coef. = 0.050, *p* = 0.092), the overall hand hygiene (Coef. = 0.027, *p* = 0.037), use of goggle (Coef. = 0.149, *p* = 0.002), use of gown (Coef. = 0.133, *p* = 0.001) and overall use of PPE (Coef. = 0.087, *p* < 0.001). Besides, the HCWs in affected area were associated with higher self-reported compliance of hand hygiene after touching patient surroundings (Coef. = 0.047, *p* = 0.062), overall hand hygiene (Coef. = 0.028, *p* = 0.012), use of goggle (Coef. = 0.079, *p* = 0.052), use of gown (Coef. = 0.113, p = 0.001), overall use of PPE (Coef. = 0.046, *p* = 0.016) and terminal disinfection (Coef. = 0.054, *p* = 0.030). Nevertheless, as coefficients were negative, the experience of contact with confirmed and suspected patients decreased the self-reported compliance of IPC measures in use of goggle (Coef. = − 0.159, *p* < 0.001), use of gown (Coef. = − 0.155, *p* < 0.001) and overall use of PPE (Coef. = − 0.087, *p* < 0.001) In addition, higher work load decreased self-reported compliance of appropriate patient placement (Coef. = − 0.018, *p* = 0.029), the use of goggle (Coef. = − 0.061, *p* = 0.002; Coef. = − 0.063, *p* = 0.009), use of gown (Coef. = − 0.044, *p* = 0.017;Coef. = − 0.045, *p* = 0.029), overall use of PPE (Coef. = − 0.026, *p* = 0.017; Coef. = − 0.030, *p* = 0.009) and report to superior (Coef. = − 0.017, *p* = 0.081). In addition, the higher education degree, longer work year, female (vs. male) had significantly negative effect on the self-reported compliance, while nurses (vs. doctors) had significantly positive effect on the self-reported compliance of IPC measures (Table 4).

**Table 4:** Negative binomial regression analysis risk factors on self-reported compliance

	Outbreak risk			Extent of risk		
	Coef.	SD	*P*-value	Coef.	SD	*P*-value
***Before touching a patient***						
Outbreak risk	0.106	0.006	< 0.001***	–	–	–
Education degree	−0.024	0.013	0.062*	–	–	–
***Before aseptic procedure***						
Outbreak risk	0.036	0.003	< 0.001***	–	–	–
Age	0.004	0.002	0.071*	–	–	–
Work year	−0.004	0.002	0.041**	–	–	–
***After body fluid exposure risk***						
Outbreak risk	0.029	0.003	< 0.001***	–	–	–
Career	0.024	0.014	0.083*	–	–	–
Work year	−0.004	0.002	0.034**	–	–	–
***After touching a patient***						
Outbreak risk	0.066	0.004	< 0.001***	–	–	–
***After touching patient surroundings***						
Outbreak risk	0.096	0.005	< 0.001***	–	–	–
High-risk Department	–	–	–	0.050	0.030	0.092*
Risk of affected area	–	–	–	0.047	0.025	0.062*
Education degree	−0.029	0.012	0.017**	–	–	–
***Overall hand hygiene***						
Outbreak risk	0.070	0.003	< 0.001***	–	–	–
High-risk Department	–	–	–	0.027	0.013	0.037**
Risk of affected area	–	–	–	0.028	0.011	0.012**
***Appropriate patient placement***						
Outbreak risk	0.063	0.005	< 0.001***	–	–	–
Gender	−0.049	0.022	0.026**	−0.065	0.035	0.064*
career	0.050	0.023	0.030**	–	–	–
Work load	−0.018	0.008	0.029**	–	–	–
***Use of mask***						
Outbreak risk	0.043	0.004	< 0.001***	–	–	–
Career	0.048	0.017	0.005***	–	–	–
***Use of glove***						
Outbreak risk	0.087	0.006	< 0.001***	–	–	–
Career	0.110	0.027	< 0.001***	0.085	0.040	0.036**
***Use of goggle***						
Outbreak risk	**0.151**	0.010	< 0.001***	–	–	–
Risk of contact with confirmed and suspected patients	–	–	–	−0.159	0.041	< 0.001***
High-risk Department	–	–	–	0.149	0.047	0.002***
Risk of affected area	–	–	–	0.079	0.041	0.052*
Gender	−0.091	0.048	0.059*	−0.101	0.056	0.069*
Work load	−0.061	0.020	0.002***	−0.063	0.024	0.009**
***Use of gown***						
Outbreak risk	0.129	0.009	< 0.001***	–	–	–
Risk of contact with confirmed and suspected patients	–	–	–	−0.155	0.036	< 0.001***
High-risk Department	–	–	–	0.133	0.041	0.001***
Risk of affected area	–	–	–	0.113	0.035	0.001***
Gender	–	–	–	−0.081	0.048	0.091*
Career	0.087	0.048	0.073*	–	–	–
Work load	−0.044	0.019	0.017**	−0.045	0.021	0.029**
***Overall use of PPE***						
Outbreak risk	0.109	0.006	< 0.001***	–	–	–
Risk of contact with confirmed and suspected patients	–	–	–	−0.087	0.019	< 0.001***
High-risk Department	–	–	–	0.087	0.023	< 0.001***
Risk of affected area	–	–	–	0.046	0.019	0.016**
Gender	–	–	–	−0.056	0.026	0.033**
Career	0.080	0.031	0.010**	0.083	0.031	0.007***
Work load	−0.026	0.011	0.017**	−0.030	0.011	0.009***
***Terminal disinfection***						
Outbreak risk	0.059	0.005	< 0.001***	–	–	–
Risk of affected area	–	–	–	0.054	0.025	0.030**
Career	0.052	0.024	0.027**	–	–	–
***Report to superior***						
Outbreak risk	0.059	0.013	< 0.001***	–	–	–
Work load	−0.017	0.009	0.081*	–	–	–

Significance: ***p < 0.01, **p < 0.05, *p < 0.10

## Discussions

To our knowledge, this is the first study to investigate the level and key risk factors of IPC self-reported behaviors of HCWs at the early stage of COVID-19 outbreak, which has practical significance for optimal IPC management among broader populations under the risk of COVID-19 infection.

Overall, IPC self-reported behaviors of HCWs were basically satisfactory. HCWs reported good hand hygiene, especially after body fluid exposure and before aseptic procedure. Hand hygiene is recognized globally as a leading measure of IPC, which has been shown to be effective in decreasing the transmission of common respiratory viruses, including human coronaviruses [9, 10], and it has also been used in respond to SARS [11–13], Ebola [14], bird flu [15], and Plague [16], etc. HCWs who reported higher hand hygiene during patient care experienced a lower risk of developing SARS [11]. However, the self-reported compliance of hand hygiene before touching patients and after touching patient surroundings was relatively low as showed in previous studies [17], which may hinder the prevention and control of COVID-19. Besides, given the high transmissibility of the COVID-19 [18], appropriate patient placement was the primary measure to contain the epidemics, and a high rate of appropriate patient placement was found in this study.

The self-reported compliance of mask and glove use when caring confirmed and suspected patients was high, but the self-reported compliance of goggle and gown use were unsatisfactory, which might suggest the insufficient awareness of HCWs on controlling new epidemic. As we know, the main transmitted routes of COVID-19 were droplets and contact [19, 20], which means that PPE is crucial for controlling COVID-19. The only way to control new life-threatening epidemics at the early stage is optimal IPC behaviors and the maximal protection including masks, gloves, gowns, and eye protection [21]. Thus, the substandard use of goggle and gown would put HCWs at the risk of virus exposure and a significant risk of cross-infection in hospitals. Researchers found that lack of awareness and discomfort of the equipment may hinder PPE use [22]. For example, a survey found that gowns and goggles were tight, foggy and induced intense perspiration and visual acuity, which hindered the care for patient of HCWs [22]. In our study, most HCWs reported to superior when encountering confirmed and suspected patients**.** According to guidelines, early identification and reporting of an epidemic was important to contain the infection [9]. Reporting can be regarded as the starting point of all subsequent IPC, and 100% reporting rate should be the goal.

Not surprising, the outbreak risk promoted self-reported IPC behaviors of HCWs, especially hand hygiene before touching patients, use of goggle and gown. Previous study also found positive changes of hand hygiene and other IPC measures during or one-year after the outbreak of SARS, which means that the outbreak risk has an effective and long-term impact on the practice of IPC measures [23, 24]. As we expected, HCWs in the affected city behave better than unaffected, indicating the increasing risk affects behavior [25].

Contrary to our expected hypothesis, the increased of exposure risk to COVID-19 patients did not promote self-reported IPC behaviors. HCWs who had direct contact with confirmed and suspected COVID-19 patients even reported worse in some IPC. It may be due to short supply of resources, human deficiency and high workload, which can be explained that the subsequent lack of protective materials and human resources, and timely assistance are in urgent need in combating COVID-19.

HCWs in high-risk department reported better behaviors in some IPC than those not. Perhaps because of the daily high incidence and cross transmission of infection in high-risk departments, HCWs usually payed more attention to IPC measures. It enlightens us that daily training and practice of IPC help to cope with sudden epidemic outbreak.

HCWs with lower workload, male and nurse profession promoted IPC behavior, compared to higher workload, female and doctor profession. This revealed that increasing number of HCWs especially male and nurses to reduce workload may be an effective measure. HCWs from all over China to support Wuhan, Hubei are the appropriate measures.

This work reveals implications to improve care in a highly dynamic, resource limited pandemic setting. Firstly, it may be constructive to equip the core clinical department of COVID-19 with sufficient HCWs, especially male and nurse profession, to relieve the high work load and ensure the higher compliance of IPC measures to reduce the risk of HCAI. Secondly, it is a priority to guarantee the supply of PPE in the core clinical department of COVID-19. Thirdly, the storage of human resources and PPE, the emergent purchase flow in hospital, the encouragement of PPE production and the development of vaccine, etc. are vital in future preparedness.

The limitation of this study is that the compliance of IPC behaviors of HCWs may be overestimated, because HCWs may respond to interview questions in a way that they believe is socially acceptable rather than being completely accurate, namely “Social desirability” [26]. However, the impact of self-reported behavior on the regression result was hard to determine, comparing to directly observed, because researchers found that the influencing factors were quite different even in the same group and no statistically significant correlations were found between observed and self-reported compliance [27]. To make the self-reported compliance closer to the actual, we devoted all the staffs in research group and trained carefully, to educate HCWs that they should complete the questionnaires based on actual situation.

## Conclusions

The self-reported IPC behaviors of HCWs significantly improved after COVID-19 outbreak. HCWs who were in the affected area and in high-risk department reported IPC behavior better. But the contact with confirmed and suspected COVID-19 patients did not promote self-reported IPC behaviors, which may result from higher workload and lack of resources such as gowns. The preparedness system including storage of human resources and PPE in hospital, the emergent purchase flow should be improved. The medical assistance is in urgent need in combating COVID-19, especially human resources with male and nurse profession.

## Supplementary information

**Additional file 1.** Questionnaire on personal protection of healthcare workers during COVID-19 outbreak

## Data Availability

The data that support the findings of this study are available from surveyed local institutions but restrictions apply to the availability of these data, which were used under license for the current study, and so are not publicly available. Data are however available from the authors upon reasonable request and with permission of surveyed local institutions.

## Abbreviations

IPC: Infection prevention and control
HCWs: Healthcare workers
PHEIC: Public Health Emergency of International Concern
PPE: Personal protective equipment
