# Supplementary material for: Will healthcare workers improve infection prevention and control behaviors as COVID-19 risk emerges and increases, in China?
Source: Antimicrob Resist Infect Control. 2020 Jun 11;9:83. doi: 10.1186/s13756-020-00746-1 (PMC7289224; doi:10.1186/s13756-020-00746-1)
Supplement: Supplementary file 1 — Additional file 1. Questionnaire on personal protection of healthcare workers during COVID-19 outbreak [file 13756_2020_746_MOESM1_ESM.docx]

**Questionnaire on personal protection of healthcare workers during COVID-19 outbreak**

The suspected patient: Influenza-like cases, fever ≥38℃, accompanied by a sore throat or cough

1. Did you treat/care the confirmed or suspected COVID-19 patient?

□yes ; □no

2. The location of your hospital

□Wuhan ; □Ganzhou

3.Your department:

|  | **One recent month**  **（after COVID-19 outbreak）** | | | | | | | | | | | **One month before**  **(before COVID-19 outbreak）** | | | | | | | | | | |
| --- | --- | --- | --- | --- | --- | --- | --- | --- | --- | --- | --- | --- | --- | --- | --- | --- | --- | --- | --- | --- | --- | --- |
|  | 10 | 9 | 8 | 7 | 6 | 5 | 4 | 3 | 2 | 1 | 0 | 10 | 9 | 8 | 7 | 6 | 5 | 4 | 3 | 2 | 1 | 0 |
| 4.In 10 times of hand hygiene opportunity before direct contact with patients, I washed my hand times | □ | □ | □ | □ | □ | □ | □ | □ | □ | □ | □ | □ | □ | □ | □ | □ | □ | □ | □ | □ | □ | □ |
| 5. In 10 times of hand hygiene opportunity before aseptic operation, I washed my hand times | □ | □ | □ | □ | □ | □ | □ | □ | □ | □ | □ | □ | □ | □ | □ | □ | □ | □ | □ | □ | □ | □ |
| 6. In 10 times of hand hygiene opportunity after exposed to patient’s body fluid, I washed my hand times | □ | □ | □ | □ | □ | □ | □ | □ | □ | □ | □ | □ | □ | □ | □ | □ | □ | □ | □ | □ | □ | □ |
| 7. In 10 times of hand hygiene opportunity after direct contact with patients, I washed my hand times | □ | □ | □ | □ | □ | □ | □ | □ | □ | □ | □ | □ | □ | □ | □ | □ | □ | □ | □ | □ | □ | □ |
| 8. In 10 times of hand hygiene opportunity after exposed to patients’ surroundings, I washed my hand times | □ | □ | □ | □ | □ | □ | □ | □ | □ | □ | □ | □ | □ | □ | □ | □ | □ | □ | □ | □ | □ | □ |
| 9.In 10 times of contacting high-risk patients, I wore mask times. | □ | □ | □ | □ | □ | □ | □ | □ | □ | □ | □ | □ | □ | □ | □ | □ | □ | □ | □ | □ | □ | □ |
| 10. In 10 times of contacting high-risk patients, I wore glove times. | □ | □ | □ | □ | □ | □ | □ | □ | □ | □ | □ | □ | □ | □ | □ | □ | □ | □ | □ | □ | □ | □ |
| 11. In 10 times of contacting high-risk patients, I wore goggle times. | □ | □ | □ | □ | □ | □ | □ | □ | □ | □ | □ | □ | □ | □ | □ | □ | □ | □ | □ | □ | □ | □ |
| 12. In 10 times of contacting high-risk patients, I wore gown times. | □ | □ | □ | □ | □ | □ | □ | □ | □ | □ | □ | □ | □ | □ | □ | □ | □ | □ | □ | □ | □ | □ |
| 13. In 10 times of treating high-risk patients, my apartment gave patients single room isolation, cohorting or bed unit isolation times. | □ | □ | □ | □ | □ | □ | □ | □ | □ | □ | □ | □ | □ | □ | □ | □ | □ | □ | □ | □ | □ | □ |
| 14. In 10 times of arranging the bed used by patient, I did terminal disinfection times. | □ | □ | □ | □ | □ | □ | □ | □ | □ | □ | □ | □ | □ | □ | □ | □ | □ | □ | □ | □ | □ | □ |
| 15.In 10 times of encountering high-risk patient, I reported to superior. | □ | □ | □ | □ | □ | □ | □ | □ | □ | □ | □ | □ | □ | □ | □ | □ | □ | □ | □ | □ | □ | □ |

| **1.** **Gender** | □Male | □Female | | | | | | | | |
| --- | --- | --- | --- | --- | --- | --- | --- | --- | --- | --- |
| **2.** **Career** | □Doctor | □Nurse | | | | | | | | |
| **3.** **Age** | __________year | | | | | | | | | |
| **4.** **Work year** | __________ year | | | | | | | | | |
| **5.** **Degree** | □Below junior college | | □junior college | | □Undergraduate | | □Master | | □Doctor | |
| **6.** **Title** | □Not evaluate | □Junior | | □Intermediate | | □Deputy senior | | | | □Senior |
| **7.** **Work load** | □Very low | □Low | | □Neutral | | □High | | □Very high | | |
